# Supplementary material for: MetaRibo-Seq measures translation in microbiomes
Source: Nat Commun. 2020 Jun 29;11:3268. doi: 10.1038/s41467-020-17081-z (PMC7324362; doi:10.1038/s41467-020-17081-z)
Supplement: Supplementary file 10 — Supplementary Data 7 [file 41467_2020_17081_MOESM10_ESM.zip › File2/Confidence_High_Taxonomy/306417_out.krona.html]

Javascript must be enabled to view this page.

members
magnitude
magnitudeUnassigned
count
unassigned
taxon
rank

306417\_out

46

2
46
superkingdom

phylum
201174
46

class
46
84998

84999
46
order

family
46
84107

46
102106

SRS015794\_contig\_number\_contig-100\_8173.8173SRS050998\_contig\_number\_contig-100\_6829.6830SRS144362\_contig\_number\_20723
3
genus

species
74426
42

SRS012969\_contig\_number\_contig-100\_25659.62199SRS013951\_contig\_number\_contig-100\_851.202308SRS014923\_contig\_number\_16674SRS015431\_contig\_number\_85712SRS015578\_contig\_number\_7439SRS015782\_contig\_number\_30915SRS017103\_contig\_number\_23526SRS018351\_contig\_number\_1145SRS019068\_contig\_number\_112538SRS019161\_contig\_number\_38505SRS020328\_contig\_number\_contig-100\_264.202456SRS020869\_contig\_number\_33556SRS021948\_contig\_number\_contig-100\_25893.25893SRS023526\_contig\_number\_contig-100\_21316.105167SRS023715\_contig\_number\_7188SRS024331\_contig\_number\_contig-100\_3092.3092SRS042966\_contig\_number\_1228SRS043411\_contig\_number\_3378SRS050422\_contig\_number\_contig-100\_692.182205SRS050752\_contig\_number\_contig-100\_18035.103591SRS050941\_contig\_number\_15776SRS053398\_contig\_number\_contig-100\_18151.57026SRS055017\_contig\_number\_contig-100\_28825.131048SRS063127\_contig\_number\_2226SRS064276\_contig\_number\_contig-100\_25570.121248SRS075878\_contig\_number\_17520SRS076876\_contig\_number\_contig-100\_14971.14972SRS076929\_contig\_number\_6697SRS077335\_contig\_number\_contig-100\_540.49956SRS077502\_contig\_number\_17422SRS097889\_contig\_number\_contig-100\_10517.165058SRS098571\_contig\_number\_64118SRS098644\_contig\_number\_26171SRS103987\_contig\_number\_contig-100\_9655.176631SRS1041031\_contig\_number\_339SRS1055049\_contig\_number\_contig-100\_36486.36487SRS140492\_contig\_number\_contig-100\_223.195687SRS143070\_contig\_number\_contig-100\_2616.2616SRS144506\_contig\_number\_contig-100\_3734.252140SRS147022\_contig\_number\_contig-100\_8849.88031SRS148424\_contig\_number\_contig-100\_37327.37327SRS971275\_contig\_number\_38831

2292336
1

SRS144135\_contig\_number\_4247
species
